# Assessing mortality risk in narcolepsy: Evidence from population studies

**DOI:** 10.1002/pcn5.70411

**Published:** 2026-09-07

**Authors:** Nawris Alhassan, Susmin Karki, Jagdish Lal

**Affiliations:** ^1^ Biomedical Sciences, Hope Clinic Midvale Utah USA; ^2^ Maharajgunj Medical Campus, Institute of Medicine Tribhuvan University Maharajgunj Nepal; ^3^ Physical & Occupational Rehabilitation Burnaby Chronic Pain & Rehabilitation Clinic Burnaby Canada

**Keywords:** meta‐analysis, mortality, narcolepsy, risk factor, systematic review

## Abstract

Narcolepsy is a chronic neurologic sleep disorder associated with excessive daytime sleepiness, cataplexy, and a higher prevalence of cardiovascular and psychiatric comorbidities. While these factors may predispose individuals to adverse events, observational studies evaluating mortality in narcolepsy have yielded inconsistent results. This systematic review and meta‐analysis aimed to quantify the all‐cause mortality risk in patients with narcolepsy compared to matched controls. A systematic literature search of PubMed, Embase, Scopus, and Web of Science was conducted up to February 2026 for studies reporting quantitative mortality metrics (Risk Ratios [RR] and Hazard Ratios [HR]) in narcolepsy. Pooled effect estimates were calculated using random‐ and fixed‐effects models. We also performed sensitivity analyses and subgroup analyses based on geographic region (Western vs. Asian) and age (Pediatric vs. Adult/Mixed) to explore heterogeneity. Five studies comprising 212,596 patients with narcolepsy and 526,688,615 controls were included in the primary analysis. The overall pooled RR for mortality was 1.25 (95% CI: 0.98 to 1.61; *P* = 0.08), demonstrating no statistically significant increase in risk, albeit with substantial heterogeneity (*I* ^2^ = 83%). A sensitivity analysis excluding one heavily weighted outlier (Ohayon et al., 2014) reduced heterogeneity (*I* ^2^ = 8%; RR = 1.09, 95% CI: 0.95 to 1.24; *P* = 0.22), showing that the results depend heavily on a single administrative dataset rather than proving narcolepsy carries no mortality risk. Furthermore, the pooled HR for mortality over time across three studies was 1.20 (95% CI: 0.82 to 1.76; *P* = 0.34). Subgroup analyses revealed no significant variation in mortality risk between Western and Asian populations (*P* = 0.55) or between pediatric and adult cohorts (*P* = 0.36). Current evidence does not prove narcolepsy carries a normal life expectancy. Instead, all‐cause mortality risk remains uncertain due to fragile data and a lack of cause‐specific findings. Because narcolepsy involves serious cardiometabolic, psychiatric, accident, and medication risks, ongoing patient risk management is required.

## INTRODUCTION

Narcolepsy is a chronic neurological sleep disorder characterized primarily by excessive daytime sleepiness (EDS) and abnormal rapid eye movement (REM) sleep regulation. Core clinical features include cataplexy, sleep paralysis, hypnagogic or hypnopompic hallucinations, and disrupted nocturnal sleep.^1^, ^2^, ^3^, ^4^, ^5^ The disorder is broadly classified into narcolepsy type 1 (NT1), which is associated with cataplexy and hypocretin deficiency, and narcolepsy type 2 (NT2), which lacks cataplexy and has a less clearly defined pathophysiology.^1^, ^4^, ^5^, ^6^, ^7^

Globally, narcolepsy is rare, with prevalence estimates ranging from 19.1/100,000 for NT1 and 23.3/100,000 for NT2 in large population studies across North America, Europe, and South Korea.^2^ Incidence estimates also vary widely, from 0.06 to 6.56 per 100,000 person‐years.^8^ The highest prevalence is observed in younger adults for NT1 and in middle‐aged adults for NT2.^2^

Several mechanisms have been proposed that may increase mortality risk in individuals with narcolepsy. EDS may increase the risk of motor vehicle accidents and occupational injuries, while cataplexy may predispose individuals to trauma and falls. In addition, narcolepsy is associated with higher rates of cardiometabolic conditions such as obesity, hypertension, and dyslipidemia, as well as psychiatric disorders including depression and suicidality, all of which may contribute to increased mortality.^1^, ^2^, ^6^, ^7^

Observational studies on mortality in narcolepsy have yielded inconsistent results. Some report elevated all‐cause mortality, while others find no significant difference compared to matched controls.^9^ Data on mortality risk across different population groups (age, sex, narcolepsy subtype) are sparse and inconclusive.^9^, ^10^ However, existing studies are limited by inconsistent findings, heterogeneous populations, and a lack of pooled quantitative synthesis, leaving the true mortality risk uncertain. Therefore, we conducted a systematic review and meta‐analysis to evaluate the association between narcolepsy and all‐cause mortality and to provide a pooled estimate of mortality risk using available population‐based studies.

## METHODS

### Protocol and registration

This systematic review and meta‐analysis were conducted and reported in accordance with the Preferred Reporting Items for Systematic Reviews and Meta‐Analysis (PRISMA) guidelines.^11^ Institutional Review Board approval or informed patient consent was waived for this review. The review protocol was registered in the PROSPERO database (CRD420251274044). This review aligns with the AMSTAR (Assessing the methodological quality of systematic reviews) guidelines ^12^ and the TITAN guidelines concerning the use of artificial intelligence.^13^ The study protocol was designed to evaluate the risk of all‐cause mortality in patients diagnosed with narcolepsy compared to matched control populations.

### Search strategy and selection criteria

A systematic literature search was performed in PubMed, Embase, Scopus, and Web of Science from database inception through February 2026. Search terms included combinations of “narcolepsy,” “sleep disorder,” “mortality,” “death,” and “survival” using Boolean operators. The reference lists of all retrieved articles and relevant review papers were also manually screened to identify any additional eligible studies. The complete, database‐specific search strings and Boolean syntax utilized for this review are reported in full in Supplementary Material 1.

### Inclusion and exclusion criteria

Studies were considered eligible for inclusion if they met the following criteria:

(1) Observational cohort or case‐control studies;
(2) Included patients with a clinically confirmed diagnosis of narcolepsy;
(3) Included a comparator group of healthy or matched individuals from the general population; and
(4) Reported quantitative data on all‐cause mortality, specifically providing Risk Ratios (RR), Hazard Ratios (HR), or sufficient raw event data to calculate these metrics.

Studies were excluded if they were case reports, case series, narrative reviews, animal studies, or if they lacked a proper control group.

### Handling of overlapping cohorts

During the selection process, we identified several studies (Jennum et al., 2013; Jennum et al., 2016; Jennum et al., 2017; Jennum et al., 2020) that drew patient data from the same source: the Danish National Patient Registry. To adhere to rigorous meta‐analytical standards and prevent the artificial inflation of statistical weight and sample size (double‐counting), we applied a strict inclusion strategy. The most comprehensive and temporally appropriate dataset was selected for the primary overall mortality analysis. The remaining overlapping studies were strictly excluded from the primary pooled analyses and were utilized *only* for specific, mutually exclusive subgroup analyses (such as comparing pediatric versus adult/mixed cohorts) where patient overlap would not compromise the pooled effect estimate.

To address overlapping registry cohorts and clarify dataset contributions across primary, sensitivity, geographic, and age‐stratified syntheses, a study‐level analytical matrix was established (Table 1).

**TABLE 1:** Analytical contribution matrix across primary, sensitivity, and subgroup syntheses.

Study	Primary RR synthesis (*N* = 5)	Sensitivity RR (excluding Ohayon) (*N* = 4)	Primary HR sysnthesis (*N* = 3)	Geographic subgroup	Age subgroup	Reason for inclusion/exclusion in primary pool
Ohayon et al. (2014)	Included	Excluded	Excluded	Western	Adult/Mixed	Primary US claims cohort; excluded in sensitivity due to single‐claim design and extreme weight.
Jennum et al. (2013)	Excluded	Excluded	Excluded	N/A	N/A	Excluded from the primary pool due to Danish registry overlap with Jennum (2017).
Jennum et al. (2016)	Excluded	Excluded	Excluded	N/A	Pediatric	Excluded from primary pool due to overlap; used qualitatively for pediatric morbidity.
Jennum et al. (2017)	Included	Included	Included	Western	Adult/Mixed	Primary Danish adult registry update.
Jennum et al. (2020)	Included	Included	Included	Western	Pediatric	Primary Danish pediatric registry update.
Lipford et al. (2024)	Included	Included	Excluded	Western	Adult/Mixed	Contributes RR data via the US electronic health record cohort.
Hsu et al. (2025)	Included	Included	Included	Asian (Taiwan)	Adult/Mixed	Primary Taiwan national health insurance cohort.

### Data extraction and quality assessment

Two independent reviewers extracted data from the included studies using a standardized extraction template. The variables extracted included: first author, publication year, country of origin, study design, follow‐up duration, participant demographics (sample size, mean age, sex distribution), and effect estimates (RR and HR) with their corresponding 95% confidence intervals (CIs) for mortality. Discrepancies between reviewers during data extraction were resolved through consensus or consultation with a third independent reviewer.

The methodological quality and risk of bias for the included observational studies were assessed using the Newcastle‐Ottawa Scale (NOS).^14^ Studies were assessed across three domains: selection of the study groups, comparability of the cohorts, and the adequacy of outcome ascertainment. Studies scoring ≥7 points were considered to be of high methodological quality.

### Statistical analysis

All statistical analyses were performed using Review Manager (RevMan) version 5.4. The primary outcomes were the pooled Risk Ratio (RR) and Hazard Ratio (HR) for all‐cause mortality, calculated with corresponding 95% CIs.

Because hazard ratios represent time‐to‐event outcomes derived from survival analyses and differ conceptually from cumulative risk measures such as risk ratios, HRs and RRs were analyzed separately in the meta‐analysis. When both adjusted and unadjusted estimates were reported, adjusted estimates were preferentially extracted to minimize residual confounding from baseline sociodemographic factors and comorbidities. Unadjusted or age/sex‐standardized rates were extracted only when multivariable models were absent in the primary study.

Specifically, extracted mortality metrics and their respective adjustment strategies were categorized as follows:

- **Jennum et al. (2017):** Extracted time‐to‐event HR for elderly cohorts (1:4 matched on age, sex, municipality, and calendar year) adjusted for educational level, marital status, employment status, and baseline psychiatric/somatic comorbidities.
- **Jennum et al. (2020):** Extracted time‐to‐event HR for pediatric/younger cohorts (1:4 matched on birth year, sex, and municipality) adjusted for parental educational level at index date.
- **Wei Hsu et al. (2025):** Extracted time‐to‐event HR (1:4 matched on age, sex, and index date) derived from multivariable Cox models adjusted for Charlson Comorbidity Index (CCI), monthly income, urbanization level, hypertension, diabetes, dyslipidemia, cardiovascular disease, chronic kidney disease, depression, and anxiety.
- **Lipford et al. (2024):** Extracted cumulative RR/odds derived from a 1:1 propensity‐score matched cross‐sectional EHR cohort controlled for birth year, age at first encounter, sex, race, ethnicity, EHR utilization (total diagnostic code count), and baseline mortality status.
- **Ohayon et al. (2014):** Extracted cumulative Standardized Mortality Ratio (SMR) and mortality rates derived from administrative claims, indirectly standardized against the general US commercial insurance population using age and sex controls without individual‐level comorbidity or lifestyle adjustments.

Dichotomous cumulative data (RRs/SMRs) were pooled using the Mantel‐Haenszel method, whereas time‐to‐event data (HRs) were pooled using the generic inverse variance method. Statistical heterogeneity was assessed using the $I^{2}$ statistic. For $I^{2}<50\%$, a fixed‐effects approach was employed; otherwise, between‐study variance was estimated using a random‐effects model.

To explore sources of heterogeneity and test findings' robustness, a sensitivity analysis was performed by systematically omitting highly weighted outlier studies (i.e., Ohayon et al., 2014). Predefined subgroup analyses evaluated whether mortality risk varied by geographic region (Western vs. Asian populations) or age group at onset (Pediatric vs. Adult/Mixed cohorts). Subgroup differences were assessed using a test for subgroup interactions (P<0.05 considered statistically significant). Finally, in accordance with Cochrane Handbook recommendations, funnel plots and formal tests for publication bias (e.g., Egger's test) were omitted because the total number of included studies per analysis was under 10, rendering asymmetry tests underpowered.

## RESULTS

### Study selection

**FIGURE 1:**
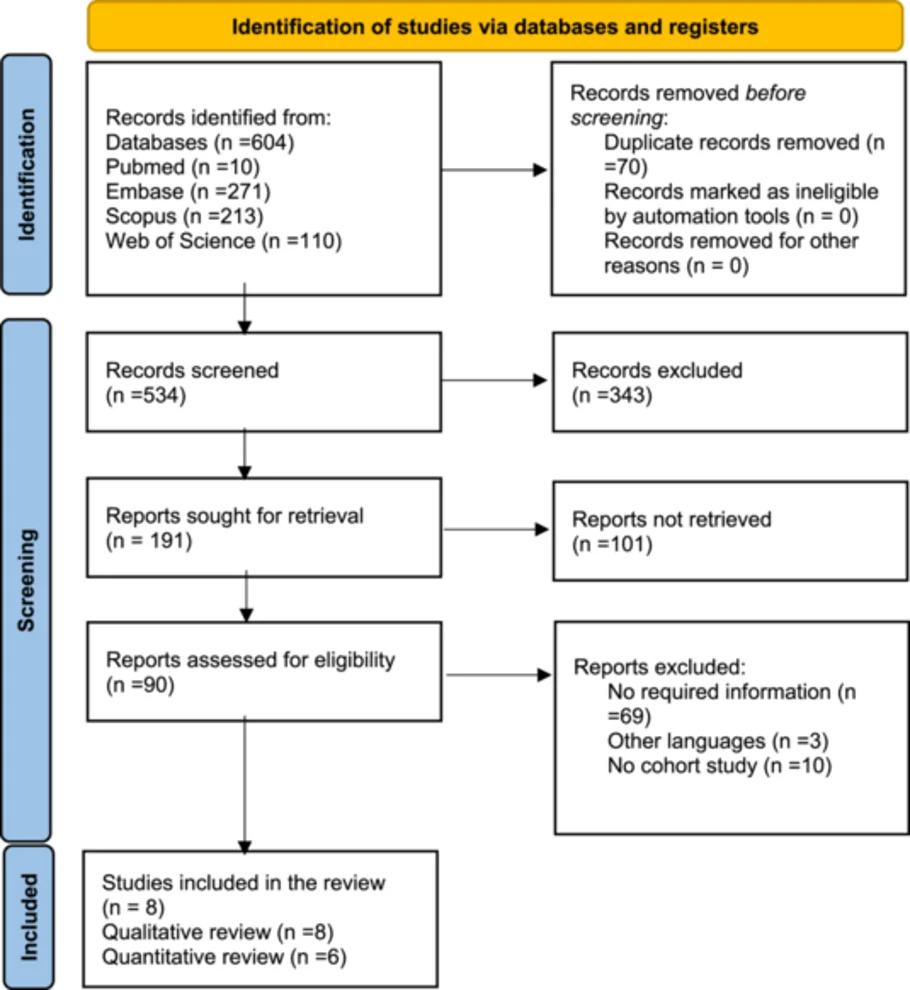
PRISMA flowchart showing the strategy for study selection. PRISMA 2020 flow diagram for new systematic reviews, which included searches of databases and registers only.

The detailed study selection process is illustrated in the PRISMA flow diagram (Figure 1). Following the application of our predefined inclusion and exclusion criteria, six distinct studies were deemed eligible for inclusion in the final review and quantitative synthesis (meta‐analysis). During this phase, two prior studies by the same research group (Jennum et al., 2013; Jennum et al., 2016) were intentionally excluded from the primary meta‐analysis to avoid data overlap from the Danish National Patient Registry, as their patient cohorts were integrated into the more comprehensive Jennum et al. (2017) and Jennum et al. (2020) updates. Instead, these two excluded studies were evaluated qualitatively within the narrative synthesis.

### Study characteristics and quality assessment

Eight distinct records met criteria for inclusion in the overall review; six provided distinct quantitative datasets across our primary and qualitative analyses, of which five contributed to the primary pooled risk ratio meta‐analysis. Geographically, the studies encompassed both Western populations (the United States and Denmark) and an Asian population (Taiwan). In total, the primary pooled analyses included 212,596 patients with a confirmed diagnosis of narcolepsy and over 526 million matched or general population controls. The mean age of the narcolepsy cohorts varied significantly depending on the study's inclusion criteria, ranging from pediatric/adolescent populations (e.g., Jennum et al., 2020) to adult and mixed‐age cohorts (e.g., Ohayon et al., 2014; Hsu et al., 2025). The detailed baseline characteristics of the included studies are presented in Table 2.

**TABLE 2:** Table showing the detailed study characteristics.

Study (Year)	Country & study design	Sample size (Narcolepsy vs. Control)	Age & follow‐up	Key morbidity, mortality & socioeconomic findings
Jennum (2020)	Denmark Prospective Registry Cohort	171 vs. 680 (1:4 matched)	Mean: 15.2 y F/U: ~20 y	Morbidity: Lower education (34% vs 52%) Mortality: HR 4.14 (Non‐sig) Socioeconomic: Lower income; higher healthcare costs (€2729 vs. €626)
Lipford (2024)	USA Propensity‐Matched Cohort (EHR/NLP)	2057 vs. 2057 (1:1 matched)	Median: 32 y F/U: Cross‐sectional	Morbidity: High mood disorders (Depression OR 3.40; Anxiety OR 2.76); CV Disease (HTN OR 1.40; Lipids OR 1.62)
Cohen (2018)	USA Community‐based Cohort	116 vs 232 Analyzed: 68 vs. 272	Mean: 30 y F/U: 8.9 y (Median)	Morbidity: OSA (OR 69.25); Obesity (OR 5.38); Anxiety (OR 4.56); Diabetes (OR 1.64). Mortality: No sig. increase in CV mortality
Jennum (2017)	Denmark National Prospective	1513 vs. 6069 (1:4 matched) Analyzed: 1020 vs. 4077)	Range: 20–59 & 60+ y F/U: Long‐term	Morbidity: Nervous system (OR 5.27); Psychiatric (OR 2.33); Respiratory (OR 2.10 in elderly) Mortality: Elderly HR 1.38 (*p* = .002) Socioeconomic: Higher social benefit usage
Jennum (2013)	Denmark National Retro/Prospective	757 vs. 3013 (1:4 matched)	All ages F/U: minimum 3 y post‐diagnosis	Morbidity: Nervous system (OR 5.27); Metabolic (OR 2.31); Musculoskeletal (OR 1.59) Mortality: HR 1.29 (Non‐sig) Socioeconomic: High annual cost (€10,039/pt)
Wei Hsu (2025)	Taiwan Retrospective Cohort	3187 vs. 12,748 (Matched + Sibling)	Mean: 24.6 y F/U: Up to 20 y	Morbidity: Suicide/Cancer (No sig. difference) Mortality: All‐cause HR 0.96 (Non‐sig); Natural Death HR 0.90; Unnatural HR 1.48
Ohayon (2014)	USA Claims Database Analysis	206,264 vs. 526,669,461 (Population dataset)	Mixed ages F/U: 3‐year window	Morbidity: Age risk highest in 35–54 bracket Mortality: Rate 0.76%–0.93% annually; SMR ~ 1.5 (Significant excess)
Jennum (2016)	Denmark National Controlled Prospective	243 vs. 970 (1:4 matched)	Range: 0–19 y F/U: Up to 2014	Morbidity: Nervous system (OR 198); Psychiatric (OR 5.8); Metabolic (OR 3.8); Pre‐diagnosis morbidity elevated Socioeconomic: High primary care utilization

*Note*: CV, cardiovascular; EHR, electronic health record; F/U, follow‐up; HR, hazard ratio; HTN, hypertension; NLP, natural language processing; OR, odds ratio; OSA, obstructive sleep apnea; SMR, standardized mortality ratio; T1/T2, Type 1/Type 2.

Methodological quality, as assessed by the NOS, was generally high across all included studies, with scores ranging from 8 to 9 out of 9 possible stars (Table 3). The studies demonstrated a low risk of selection and attrition bias, primarily due to the utilization of robust, national‐level health registries and large electronic health record databases for both exposure and outcome ascertainment. Minor risks of bias were noted in the “Comparability” domain for studies that could not fully adjust for individual‐level lifestyle factors, such as body mass index (BMI) or smoking status.

**TABLE 3:** Risk of bias assessment for cohort studies using the Newcastle Ottawa Scale (NOS).

Study	Selection (⋆⋆⋆⋆)	Comparability (⋆⋆)	Outcome (⋆⋆⋆)	Total stars	Quality rating
**Jennum (2017)**	★★★★	★★	★★★	9	High
**Jennum (2020)**	★★★★	★★	★★★	9	High
**Ohayon (2014)**	★★★★	★	★★★	8	High
**Cohen (2018)**	★★★	★★	★★★	8	High
**Lipford (2024)**	★★★★	★★	★★	8	High
**Wei Hsu (2025)**	★★★★	★★	★★★	9	High

### Overall mortality risk (risk ratio analysis)

**FIGURE 2:**
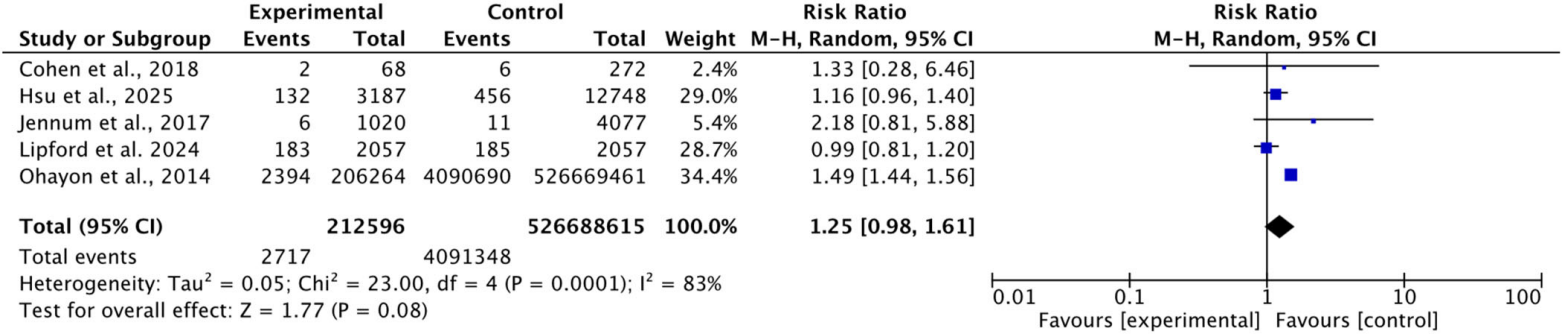
Forest plot displaying the pooled risk ratio (RR) of all‐cause mortality among patients with narcolepsy compared to controls. The analysis utilizes a Mantel–Haenszel (M–H) random‐effects model with 95% confidence intervals (CIs).

A meta‐analysis of five studies evaluating the risk of mortality in patients with narcolepsy compared to a control group was performed. Across all studies, which included 212,596 patients with narcolepsy and 526,688,615 controls, the pooled risk ratio (RR) was 1.25 (95% CI: 0.98 to 1.61). Because the confidence interval crosses 1.0 and the overall test for effect is not statistically significant (*P* = 0.08), the combined data do not demonstrate a definitively higher mortality risk in the narcolepsy group. Furthermore, there is substantial heterogeneity among the included studies (*I* ^2^ = 83%, *P *< 0.0001). For example, while Ohayon et al. (2014) carries the most weight (34.4%) and indicates a statistically significant increased risk (RR = 1.49, 95% CI: 1.44 to 1.56), other heavily weighted studies such as Lipford et al. (2024) and Hsu et al. (2025) report risk ratios closer to the line of no effect (RR = 0.99 and 1.16, respectively) that do not individually reach statistical significance (Figure 2).

**FIGURE 3:**
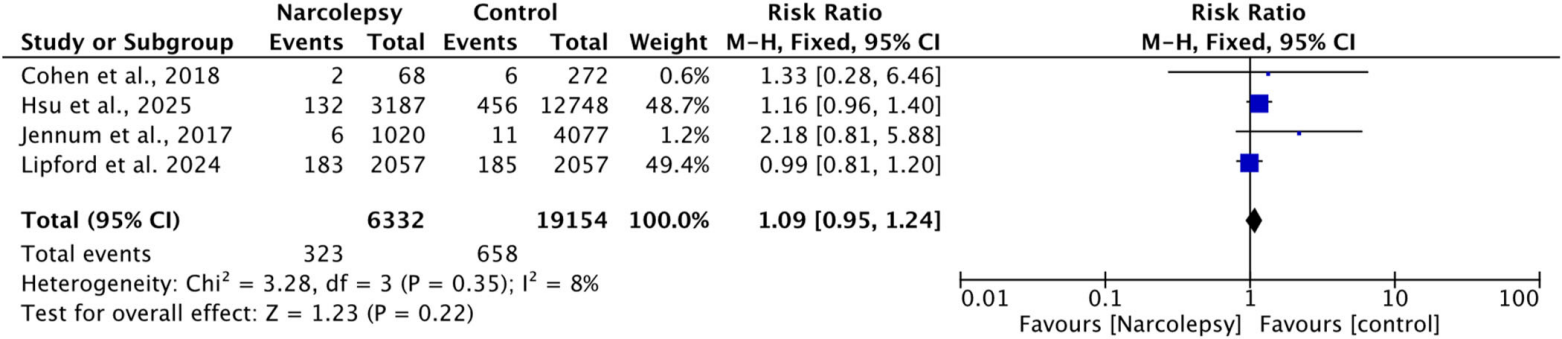
Sensitivity analysis forest plot displaying the risk ratio (RR) of mortality among patients with narcolepsy, excluding the highly weighted Ohayon et al. study. The analysis utilizes a Mantel–Haenszel (M–H) fixed‐effects model with 95% CIs.

A sensitivity analysis was conducted by excluding the heavily weighted Ohayon et al. (2014) study to assess its impact on the overall meta‐analysis. While the exclusion of this study is statistically defensible and resulted in a sharp decline in *I* ^2^ from 83% down to 8% (*P* = 0.22), this dramatic drop should be interpreted not as a true resolution of underlying clinical heterogeneity, but rather as clear evidence that the overall meta‐analytic conclusions are highly sensitive to this single dataset. In this updated fixed‐effects model excluding the Ohayon dataset, the analysis of the remaining four studies, comprising 6,332 narcolepsy patients and 19,154 controls, yielded a pooled risk ratio of 1.09 (95% CI: 0.95–1.24). With Ohayon removed, almost all the statistical weight shifted to Hsu et al. (48.7%) and Lipford et al. (49.4%). The overall test for effect remains statistically non‐significant (*P* = 0.22). Rather than confirming an absence of risk, the pooled estimate shifted toward the line of no effect, and the pooled estimate is heavily dependent on the inclusion or exclusion of a single large administrative claim dataset, reflecting high statistical fragility (Figure 3).‬‬‬‬‬‬

### Mortality hazard over time (hazard ratio analysis)

**FIGURE 4:**
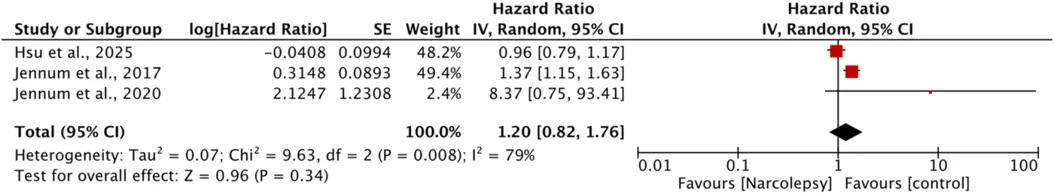
Forest plot displaying the pooled hazard ratio (HR) of mortality among patients with narcolepsy compared to controls. Data were pooled using a generic inverse variance (IV) random‐effects model with 95% CIs.

In addition to the risk ratio calculations, we pooled data from three studies reporting hazard ratios (HR) to further evaluate the mortality risk associated with narcolepsy over time. The random‐effects meta‐analysis yielded an overall pooled HR of 1.20 (95% CI: 0.82 to 1.76). Consistent with the primary risk ratio findings, this result indicates no statistically significant difference in mortality hazard between the narcolepsy and control groups (*P* = 0.34). Furthermore, substantial heterogeneity was observed among these three studies (*I* ^2^ = 79%, *P* = 0.008). This inconsistency is primarily driven by the conflicting results of the two most heavily weighted studies: while Jennum et al. (2017) reported a statistically significant increase in mortality hazard for patients with narcolepsy (HR = 1.37, 95% CI: 1.15 to 1.63), Hsu et al. (2025) found a hazard ratio close to the null with no significant effect (HR = 0.96, 95% CI: 0.79 to 1.17). A third study, Jennum et al. (2020), reported a much higher but non‐significant point estimate (HR = 8.37) with a very wide confidence interval, contributing minimal weight (2.4%) to the overall pooled effect (Figure 4).

### Subgroup analysis

#### Western vs. Asian populations

**FIGURE 5:**
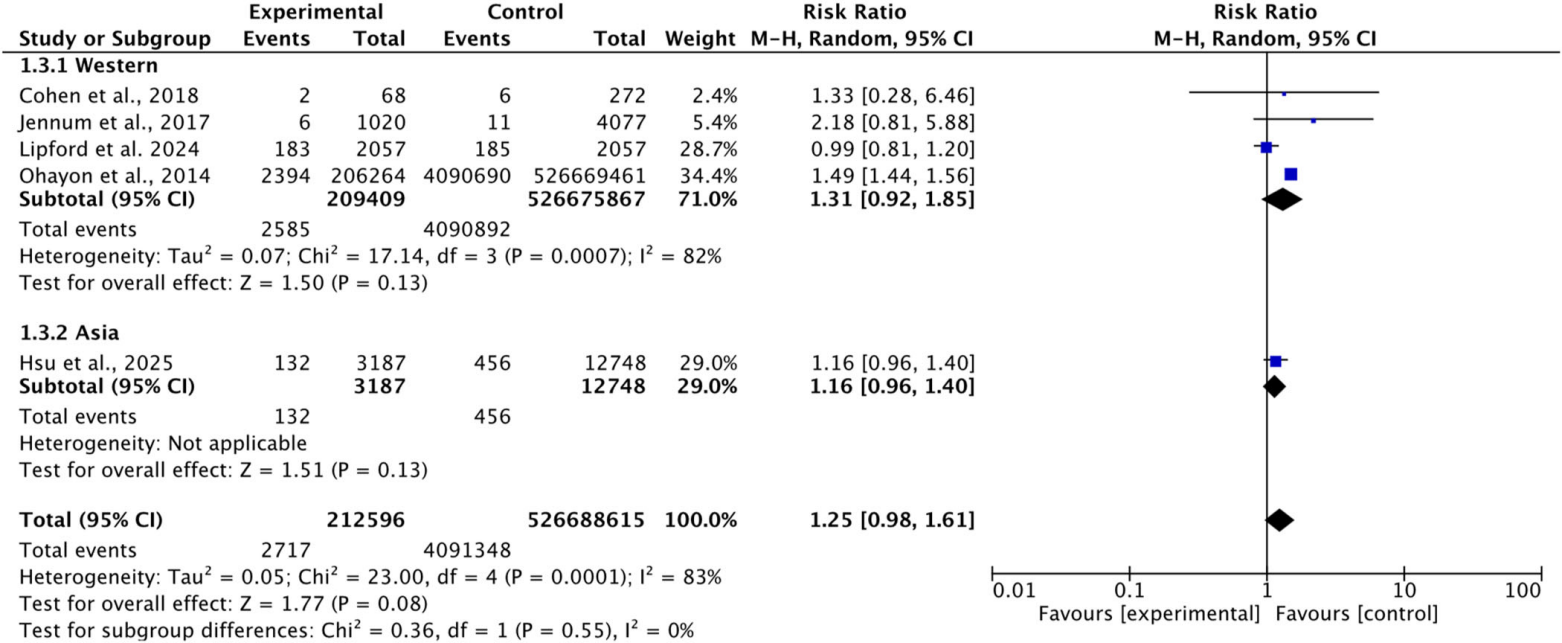
Subgroup analysis forest plot comparing the risk ratio (RR) of mortality between Western and Asian cohorts of narcolepsy patients. The analysis was conducted using a Mantel–Haenszel (M–H) random‐effects model with 95% CIs.

A subgroup analysis was conducted to evaluate whether the risk of mortality in patients with narcolepsy compared to controls differs between Western and Asian populations. The Western subgroup, which included four studies, yielded a pooled risk ratio (RR) of 1.31 (95% CI: 0.92 to 1.85). This indicates a trend toward increased mortality, though it did not reach statistical significance, and the data showed substantial heterogeneity (*I* ^2^ = 82%). The Asian subgroup, represented by a single study (Hsu et al., 2025), reported a similarly non‐significant RR of 1.16 (95% CI: 0.96 to 1.40); therefore, the pooled estimate represents the effect reported in that study rather than a true meta‐analytic synthesis. Importantly, the test for subgroup differences revealed no statistically significant distinction between the Western and Asian cohorts (*P* = 0.55). Because the Asian subgroup is represented by a single Taiwanese cohort (Hsu et al., 2025), this result reflects single‐country data rather than a true pooled continent‐wide estimate. Given this limitation, geographic subgroup comparisons are underpowered and must be interpreted with extreme caution. Overall, the combined risk ratio across all studies was 1.25 (95% CI: 0.98 to 1.61), which also narrowly missed statistical significance (*P* = 0.08) (Figure 5).

**FIGURE 6:**
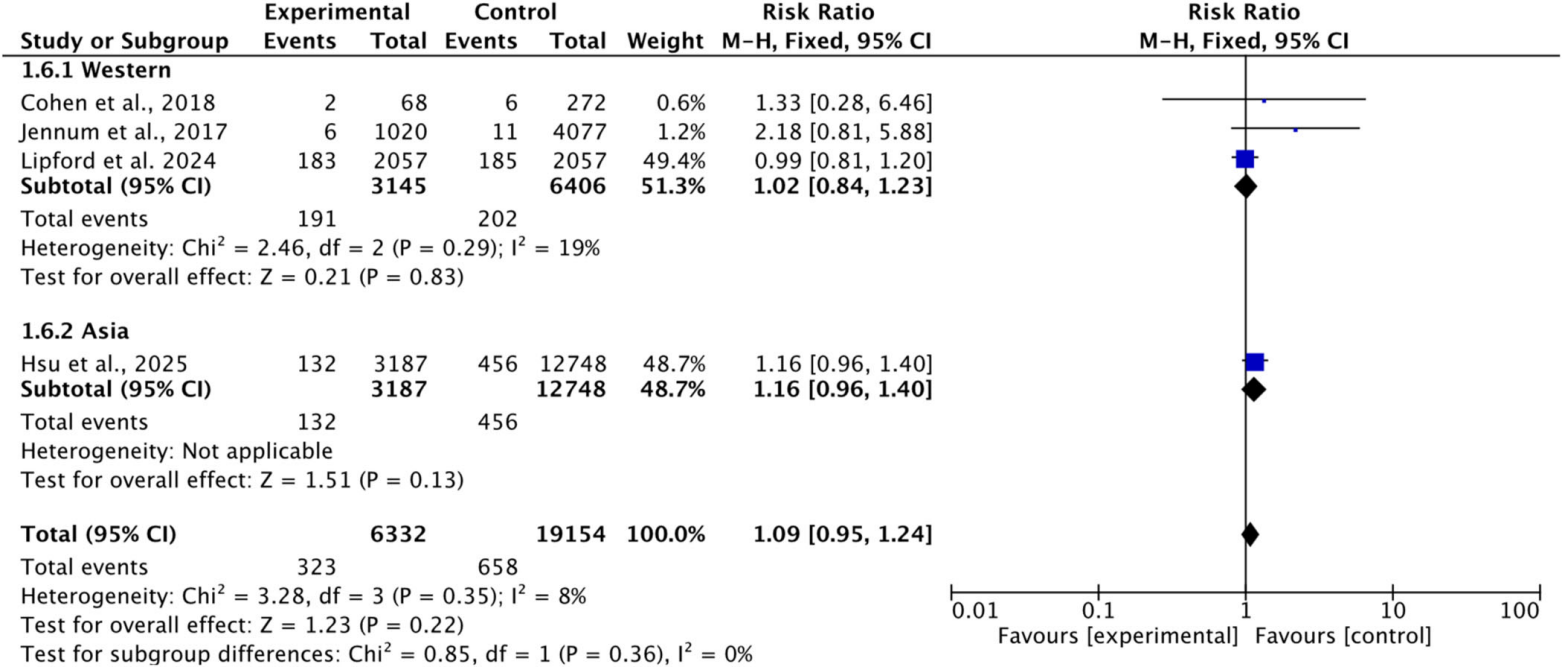
Subgroup analysis forest plot comparing the risk ratio (RR) of mortality between Western and Asian cohorts (excluding the Ohayon et al. study). The analysis was conducted using a Mantel–Haenszel (M–H) fixed‐effects model with 95% CIs.

When stratified by geographic region, the “Western excluding Ohayon” subgroup comprising three studies showed an RR of 1.02 (95% CI: 0.84 to 1.23), indicating mortality rates virtually identical to those of controls. The Asian subgroup, represented by the single study by Hsu et al. (2025), similarly reported a non‐significant RR of 1.16 (95% CI: 0.96 to 1.40). Notably, the test for subgroup differences confirmed that there is no statistically significant variance in mortality risk between the Western and Asian cohorts (*P* = 0.36, *I* ^2^ = 0%), suggesting that geographic population does not modify the overall finding of no significantly elevated mortality risk in this dataset (Figure 6).

#### Pediatric vs. adult/mixed cohorts

**FIGURE 7:**
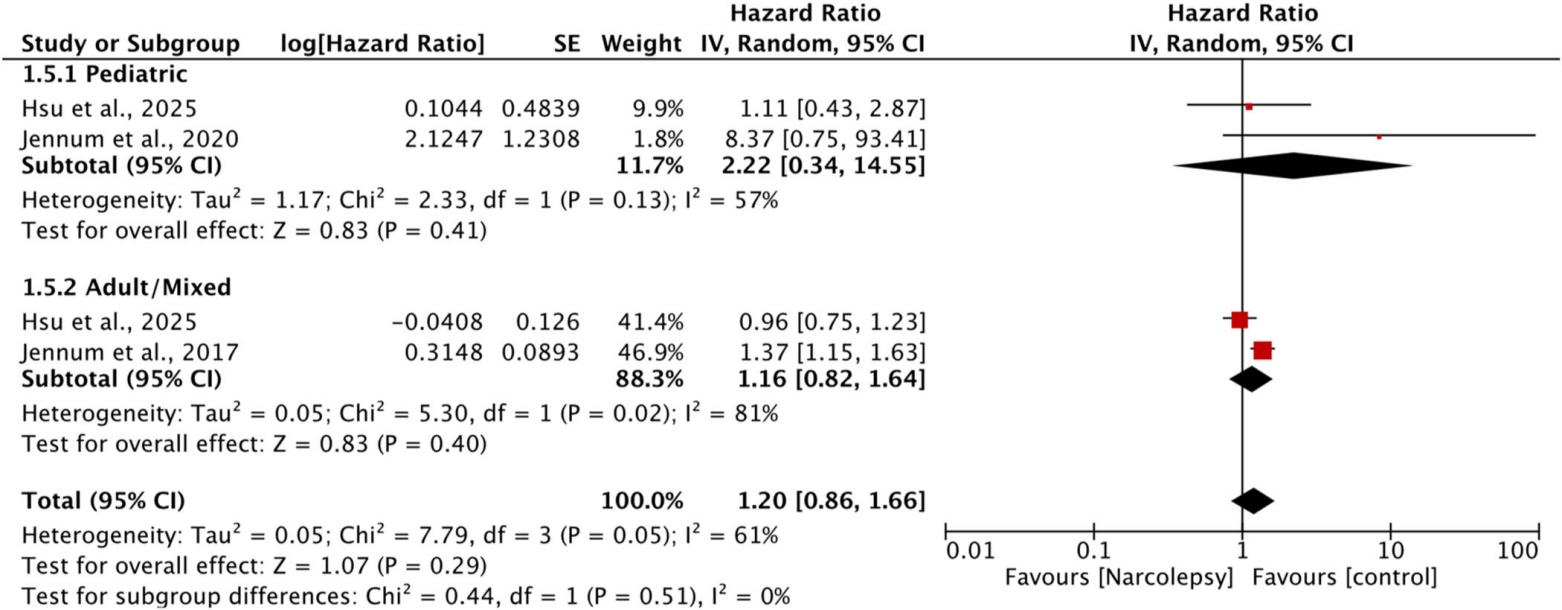
Subgroup analysis forest plot comparing the hazard ratio (HR) of mortality between pediatric and adult/mixed narcolepsy cohorts. The analysis utilizes a generic inverse variance (IV) random‐effects model with 95% CIs.

A further subgroup analysis was conducted using hazard ratios (HR) to evaluate whether the risk of mortality differs by age group, specifically comparing pediatric and adult/mixed narcolepsy cohorts. The pediatric subgroup, comprising two study populations, yielded a pooled HR of 2.22 (95% CI: 0.34–14.55). Despite the elevated point estimate, this result was not statistically significant (*P* = 0.41) due to wide confidence intervals, and it exhibited moderate heterogeneity (*I* ^2^ = 57%). The adult/mixed subgroup, which carried the vast majority of the statistical weight (88.3%), demonstrated a pooled HR of 1.16 (95% CI: 0.82 to 1.64). This was also non‐significant (*P* = 0.40) but showed high heterogeneity (*I* ^2^ = 81%). Importantly, the test for subgroup differences indicated no statistically significant variance between the pediatric and adult/mixed cohorts (*P* = 0.51, *I* ^2^ = 0%). The pediatric subgroup estimate yielded an extremely wide confidence interval due to the extremely small number of recorded deaths. This analysis is heavily underpowered, precluding any definitive conclusions regarding age‐stratified mortality risk (Figure 7).

### Qualitative analysis

**Jennum (2020):** This prospective registry study investigated the long‐term health and socioeconomic outcomes of 171 Danish children and adolescents with narcolepsy compared to 680 matched controls. Regarding mortality, the study recorded one death in the narcolepsy group and one in the control group. While the study estimated a high Hazard Ratio for mortality (HR 8.37), the result was not statistically significant (95% CI: 0.25–280.40, *p* = 0.231) due to the rarity of mortality events in this young population. This finding highlights that while a trend toward increased risk may exist, childhood‐onset narcolepsy does not show a statistically significant impact on survival within the first two decades of life.

**Lipford (2024):** Utilizing aggregate electronic health record data and natural language processing (NLP), this study characterized a large US cohort of 2057 patients with narcolepsy and 2057 propensity‐matched controls. While the primary focus was on the “complex phenotype” and symptoms like mood disorders and cardiovascular risk (OR 1.40 for hypertension), the study provides foundational data for mortality risk by identifying a significantly higher prevalence of life‐shortening comorbidities. Although specific mortality rates were not the primary endpoint of this NLP‐based characterization, the authors suggest that the increased burden of metabolic and cardiovascular disease identified via NLP provides a clinical basis for the elevated mortality observed in other longitudinal narcolepsy studies.

**Cohen (2018):** This community‐based study followed 116 narcolepsy patients and 232 controls from Olmsted County, MN, to assess comorbidities at diagnosis and during follow‐up. During a median follow‐up of 8.9 years, the study investigated whether narcolepsy led to an increase in new‐onset cardiovascular disease or death. The authors found that despite high rates of obesity (OR 5.38) and OSA (OR 69.25), there was no statistically significant increase in new‐onset cardiovascular mortality in this specific community sample compared to controls. This suggests that in a community setting with high levels of care, the short‐to‐medium‐term mortality risk may be mitigated.

**Jennum (2017):** This study specifically examined mortality and morbidity across different age groups, comparing 1513 Danish patients with 6069 controls. The results revealed a significant age‐dependent risk for mortality: elderly patients (60+ years) had a significantly higher hazard ratio for death (HR 1.38; 95% CI: 1.12–1.69; *p* = 0.002). In the middle‐aged group (20–59 years), a similar but statistically non‐significant trend toward increased mortality was observed (HR 1.35; 95% CI: 0.94–1.95; *p* = 0.106). These findings confirm that narcolepsy is associated with excess mortality, particularly in older populations where the cumulative burden of disease is higher. The study reported age‐stratified results; we performed a fixed‐effects inverse‐variance meta‐analysis to pool the hazard ratios of the middle‐aged (20–59 years) and elderly (60+ years) cohorts into a single estimate for the adult population (HR = 1.37, 95% CI: 1.15–1.64).

**Jennum (2013):** In a national retro‐ and prospective study of 757 Danish patients and 3013 controls, the researchers analyzed survival rates alongside morbidity. The study found a Hazard Ratio for mortality of 1.29 (95% CI: 0.85–1.97) for patients with narcolepsy. While this indicated a 29% higher risk of death compared to the general population, the result was not statistically significant in this all‐ages cohort. The authors noted that the increased mortality risk is likely linked to the high prevalence of endocrine and metabolic comorbidities, which were significantly elevated (OR 2.31) following the diagnosis.

**Wei Hsu (2025):** This retrospective cohort study provides the largest Asian dataset, analyzing 3187 Taiwanese patients against 12,748 population controls and a sibling cohort. The analysis found that narcolepsy was not associated with an increased risk of all‐cause mortality (HR 0.96; 95% CI: 0.79–1.17) or natural‐cause mortality (HR 0.90; 95% CI: 0.73–1.11). Interestingly, there was a trend toward higher mortality from unnatural causes (HR 1.48; 95% CI: 0.88–2.48), though it did not reach significance. This study is critical to the meta‐analysis as it contradicts the increased mortality found in Western cohorts, suggesting ethnic or healthcare system differences.

**Ohayon (2014):** Using a US claims database of 173 million individuals, this study evaluated mortality by linking 9312 narcolepsy cases to the Social Security Death Master File. The study found that annual all‐cause mortality rates were consistently higher in the narcolepsy group (0.75%–0.93%) compared to the general population. The SMR was approximately 1.5, indicating a significant 1.5‐fold excess mortality risk. The risk was most pronounced in younger and middle‐aged adults (ages 35–54), suggesting that narcolepsy is indeed associated with a reduced lifespan in the US population.

**Jennum (2016):** This controlled national study evaluated 243 pediatric narcolepsy patients (ages 0–19) and 970 controls in Denmark. While no deaths were recorded during the specific follow‐up period for this pediatric group, the study highlighted that morbidity—a known precursor to mortality—was significantly elevated as early as three years before diagnosis. The authors concluded that the high psychiatric (OR 5.8) and metabolic (OR 3.8) burden in children creates a high‐risk health profile that may contribute to the excess mortality observed in adult‐focused longitudinal studies.

## DISCUSSION

### Main findings and comparison with existing literature

This systematic review and meta‐analysis provide a comprehensive evaluation of the mortality risk associated with narcolepsy. Our pooled analysis of over 212,000 patients and a large population‐based control dataset did not demonstrate a statistically significant increase in all‐cause mortality risk among individuals with narcolepsy (RR = 1.25, *P* = 0.09; HR = 1.20, *P* = 0.34). However, substantial heterogeneity was observed across studies. This variability likely reflects differences in study design, population characteristics, and adjustment for confounders across studies. These findings highlight that the current observational estimate of all‐cause mortality risk in narcolepsy remains uncertain and highly dependent on study methodology. Rather than proving a normal life expectancy, the statistical shift observed upon omitting single‐claim administrative data demonstrates model instability, necessitating ongoing clinical monitoring for cardiovascular, metabolic, and traumatic liabilities.

Interpreting mortality estimates across the narcolepsy literature requires careful consideration of heterogeneous adjustment strategies, baseline covariates, and estimand definitions. Claims‐based cumulative risk assessments (e.g., Ohayon et al., 2014) rely on population‐level age and sex standardization without adjusting for critical individual confounders such as BMI, smoking status, or underlying cardiometabolic burden. Conversely, longitudinal population registry studies (e.g., Wei Hsu et al., 2025; Jennum et al., 2017) supply hazard ratios derived from multivariable Cox proportional hazards or propensity‐matching models adjusting for baseline cardiovascular conditions, psychiatric comorbidities, socioeconomic position, and healthcare utilization patterns. Because HRs account for variable follow‐up durations and censoring while RRs capture cumulative incidence over fixed windows, evaluating these metrics independently is essential to avoid misinterpreting comorbidity‐driven excess mortality as an intrinsic feature of narcolepsy pathophysiology.

Furthermore, differences in control construction and healthcare system contexts are substantial; Ohayon et al. compared narcolepsy cases against a massive, unmatched general population denominator within a US commercial insurance database, which inherently reflects specific healthcare‐seeking behaviors and selection biases. Conversely, the European and Asian cohorts leveraged single‐payer, universal national registries (such as the Danish National Patient Registry and Taiwan National Health Insurance Research Database) to assemble strict, propensity‐matched or 1:4 sex‐ and age‐matched controls, alongside sibling cohorts, to minimize genetic and environmental confounding. Finally, the calendar periods and follow‐up durations fundamentally alter the nature of the survival data. Ohayon et al. analyzed a compressed, cross‐sectional three‐year window (2008–2010) with mortality linkage to the Social Security Administration Death Master File to extract annualized, crude mortality metrics. This stands in stark contrast to the true longitudinal tracking of Jennum et al. and Hsu et al., who monitored cohorts over expanded historical periods for up to 20 years of continuous time‐to‐event survival. Combined with the fact that Ohayon et al. limited comorbidity adjustments to basic age and sex standardization, failing to control for residual cardiometabolic or psychiatric confounders balanced by the other propensity‐matched studies—these architectural differences explain why their findings show a uniquely elevated mortality risk compared to the more conservative risks identified in well‐matched longitudinal registries.

Current evidence indicates that the observed mortality risk in patients with narcolepsy is generally lower than that reported for other chronic sleep disorders, such as obstructive sleep apnea (OSA) with HR ranging from 1.2 to 2 and REM sleep behavior disorder (RBD), and is more comparable to or slightly higher than that seen in chronic insomnia, depending on age and comorbidity profile. Large registry‐based cohort studies and recent systematic analyses have found that, after adjustment for comorbidities and confounders, narcolepsy is not materially associated with increased all‐cause or cause‐specific mortality in the general population. However, older adults (≥65 years) with narcolepsy may exhibit a modestly elevated risk. Some registry‐based studies suggest increased mortality in older populations, although this association is not consistently observed across all cohorts.^9^

### Pathophysiological mechanisms and comorbidities

Narcolepsy is associated with increased rates of obesity, diabetes, hypertension, and dyslipidemia, as demonstrated in meta‐analyses and large cohort studies.^15^, ^16^, ^17^, ^18^, ^19^ These comorbidities are established risk factors for cardiovascular events and mortality, and their presence may contribute to the observed excess risk in older adults or in subgroups with high comorbidity burden.^16^, ^17^, ^19^ More broadly, observational evidence across sleep disorders and population cohorts underlines how disrupted sleep rhythms and behavioral signals serve as critical indicators for long‐term health outcomes.^20^ This is particularly salient given that chronic vascular comorbidities, including heightened myocardial infarction risk^21^ and cerebrovascular complications,^22^, ^23^ represent well‐established drivers of excess population‐level mortality.

Depression and psychiatric comorbidities are also common in narcolepsy. Still, recent registry data indicate that, while suicidal ideation and depression are more frequent, there is no significant increase in suicide mortality after adjustment for comorbidities and access to care.^9^

The increased mortality risk in narcolepsy, especially type 1, is hypothesized to result from a combination of behavioral, autonomic, metabolic, and psychiatric mechanisms, many of which are directly linked to the core pathophysiology of the disorder. While these mechanisms suggest a theoretical increase in mortality, our meta‐analysis suggests that these risks may be mitigated in clinical practice. Sleep attacks and cataplexy triggered by strong emotions can lead to motor vehicle accidents and occupational injuries. Narcolepsy is also associated with reduced physical activity and a higher prevalence of psychiatric comorbidities such as depression and suicidality, which may contribute to increased mortality risk.^24^ However, recent large‐scale studies have not found a significant increase in mortality from unnatural causes (accidents, suicides) among patients with narcolepsy, likely reflecting the adoption of safety behaviors, effective treatment, and risk mitigation strategies following diagnosis.^9^ Nevertheless, these risks remain clinically relevant, particularly in untreated or undiagnosed individuals. Orexin deficiency may also contribute through autonomic dysfunction, potentially predisposing to arrhythmogenesis and sudden cardiac events.^24^ Sex‐specific and age‐specific differences in mortality have not been robustly established. However, some studies suggest a higher risk in males and in younger adults, likely reflecting greater exposure to accident risk and comorbidities.^24^ The apparent discrepancy between an increased burden of cardiometabolic and psychiatric comorbidities and the absence of a significant mortality signal may reflect improved diagnosis, treatment, and behavioral adaptation following disease recognition.

### Clinical management and treatment effects

Pharmacologic management of narcolepsy may also influence morbidity and mortality. Sodium oxybate (oxybate), approved for cataplexy and EDS, is generally well tolerated but carries risks of respiratory depression, CNS depression, and abuse potential, particularly in patients with comorbid sleep‐disordered breathing.^25^ Antidepressants, commonly used for cataplexy, can cause anticholinergic side effects, cardiovascular effects, and, if withdrawn abruptly, severe rebound cataplexy, which may increase the risk of injury.^24^ Stimulants (e.g., modafinil, amphetamines) may increase cardiovascular risk, especially in patients with underlying heart disease.^24^ The overall contribution of these medications to mortality is not fully quantified, but careful selection and monitoring are warranted. Inadequately treated narcolepsy, whether due to suboptimal medication or lack of access to care, may further increase risk, while effective treatment can mitigate some adverse outcomes, though not all risks are eliminated.^24^

Clinical management strategies should be informed by these findings. Driving restrictions and accident prevention remain essential, particularly for untreated or newly diagnosed patients, given the risk of sleep attacks and cataplexy. Cardiovascular risk monitoring is warranted due to the high prevalence of obesity, metabolic syndrome, and hypertension in narcolepsy, even if adjusted mortality risk is not significantly elevated.^9^ Routine psychiatric screening and access to mental health care are critical, as depression and suicidal ideation are common. However, suicide mortality does not appear to be increased in well‐managed cohorts. Public health and policy measures should prioritize early diagnosis programs to reduce diagnostic delay, workplace accommodations to mitigate accident risk, and driver safety laws tailored to the unique risks of narcolepsy. These interventions may further reduce morbidity and mortality, particularly in high‐risk subgroups such as older adults and those with severe comorbidities.^9^

### Methodological considerations and biases

The interpretation of mortality data in narcolepsy is complicated by several inherent biases. Survivorship bias may occur if cohorts preferentially include individuals who have survived initial high‐risk periods or who have adopted safety behaviors post‐diagnosis, thereby underestimating the true risk. Referral bias is also a factor, as tertiary care cohorts may overrepresent severe cases, while population‐based studies may underrepresent undiagnosed or milder cases.

Diagnostic delay, underdiagnosis, with median delays exceeding a decade, and misclassification of narcolepsy can substantially affect mortality risk estimates.^9^, ^18^ This may result in misattribution of deaths to comorbid conditions rather than narcolepsy, underestimation of mortality risk in registry studies, and confounding by unrecognized disease severity. Additionally, direct adjustment for BMI is often lacking in administrative data, leaving room for residual confounding by obesity, a key risk factor in this population. The granularity of adjustment for psychiatric comorbidities, such as depression severity and treatment status, is similarly limited.

### Strengths and limitations

The current evidence base examining mortality in narcolepsy has several strengths and limitations. Large population‐based registry studies with extended follow‐up periods provide robust data on all‐cause mortality, while matched controls and sibling cohorts enhance methodological rigor. An additional strength of this analysis is the inclusion of very large population‐based control cohorts, enhancing statistical precision and generalizability.

While overall cohort sizes may be substantial, the number of cause‐specific deaths (e.g., cardiovascular, accidental, suicide‐related) remains low, particularly in subgroups such as sibling controls, limiting statistical power for these outcomes and precluding definitive conclusions regarding specific mortality risks.^9^ Additionally, cause‐specific mortality (e.g., cardiovascular, accidental, or suicide‐related deaths) could not be robustly evaluated due to limited and inconsistent reporting across studies. Most available data are derived from single‐country registries (e.g., Taiwan), which may not be generalizable to populations with different healthcare systems, clinical practices, or lifestyle factors. The lack of multinational studies restricts the ability to extrapolate findings globally.^9^ Although some studies feature long‐term follow‐up, the relatively young age of narcolepsy cohorts at baseline means that late‐onset mortality related to comorbid conditions (e.g., cardiovascular disease, obesity) may be underrepresented. Longer observation periods are needed to capture the full spectrum of mortality risk.^9^ Key lifestyle and occupational factors (e.g., smoking, diet, work exposures) are often not captured, limiting adjustment for important confounders.

Additionally, objective sleep study data (polysomnography, multiple sleep latency testing) are frequently absent from administrative databases, potentially affecting diagnostic accuracy.^9^ There is heterogeneity in diagnostic criteria and limited ability to distinguish narcolepsy subtypes (type 1 vs. type 2), which complicates subgroup analyses and may obscure differential risks. Standardized reporting of outcomes and phenotypes is lacking.^9^

Second, our age‐stratified conclusions, particularly regarding pediatric populations, remain highly fragile. All‐cause mortality events in children and adolescents diagnosed with narcolepsy are exceptionally rare. This absolute sparsity of events compromises statistical power, inflates confidence intervals, and means that minor fluctuations in registry recording could heavily skew the findings. Therefore, the pediatric subgroup analysis yielded an extremely wide confidence interval due to the very low number of recorded deaths. This analysis is heavily underpowered, precluding any definitive conclusions regarding age‐stratified mortality risk. Furthermore, our geographic subgroup analysis was severely constrained by data availability. Because the Asian subgroup relied exclusively on data from Taiwan, this result represents single‐country data rather than a true pooled Asian continent estimate. Consequently, geographic subgroup comparisons are underpowered, and any comparative conclusions between Western and Eastern cohorts must be substantially scaled back until multi‐country Asian data emerge.

### Future research and clinical implications

To address these critical gaps, future research should prioritize the establishment of international, multicenter registries to enhance geographic diversity and generalizability. Long‐term prospective cohort studies are needed, incorporating comprehensive clinical phenotyping, standardized outcome definitions, and detailed confounder assessments (including lifestyle factors, BMI, and treatment exposures). The continued use of sibling or family‐based controls will be vital to further mitigate confounding by genetic and early environmental factors. Finally, evaluating cause‐specific mortality is essential, as all‐cause mortality may obscure important differences in risk profiles; analyses of specific causes, such as accidental or suicide‐related deaths, will yield actionable insights to inform targeted prevention strategies and improve patient outcomes. Future research should prioritize large, multinational prospective cohorts with detailed phenotyping and cause‐specific mortality analysis to better define risk profiles in specific patient subgroups.

## CONCLUSION

In conclusion, this exploratory meta‐analysis and narrative synthesis highlight the structural fragmentation and methodological limitations within the current global evidence base on narcolepsy mortality. Rather than providing a definitive quantitative verdict, the principal contribution of this study is a clear call to action where the field must transition away from isolated database extractions and toward large‐scale, standardized international registries and collaborative patient‐level data analyses utilizing harmonized clinical and adjustment frameworks.

Clinically, fluctuating statistical significance must not dictate real‐world patient care. Current evidence does not prove a normal life expectancy; rather, it supports an uncertain all‐cause mortality risk characterized by high statistical heterogeneity, model fragility, and a critical lack of cause‐specific mortality data. Because narcolepsy remains a complex systemic condition tied to established cardiometabolic, psychiatric, accident, and medication‐related liabilities, continuous comorbidity monitoring and proactive risk mitigation remain universally warranted for all practicing physicians, completely independent of mathematical thresholds.

## AUTHOR CONTRIBUTIONS

**Nawris Alhassan, Susmin Karki,** and **Jagdish Lal:** Conceptualization; data collection; writing—original draft; manuscript revision.

## CONFLICT OF INTEREST STATEMENT

The authors declare no conflicts of interest.

## ETHICS STATEMENT

This article is a systematic review of published literature and does not contain any studies with human participants or animals performed by any of the authors. Ethics approval was not required for this systematic review.

## INFORMED CONSENT

Informed consent was not required for this systematic review.

## PEER AND PROVENANCE STATEMENT

Not commissioned, externally peer‐reviewed.

## Supporting information

Supporting file 1.

Supporting file 2.

## Data Availability

The data that support the findings of this study are available from the corresponding author upon reasonable request.
