# Supplementary material for: Assessing mortality risk in narcolepsy: Evidence from population studies
Source: PCN Rep. 2026 Sep 7;5(3):e70411. doi: 10.1002/pcn5.70411 (PMC13549222; doi:10.1002/pcn5.70411)
Supplement: Supplementary file 1 [file PCN5-5-e70411-s001.docx]

**Supplementary Material 1: Detailed Literature Search Strategy**

**Protocol and Reproducibility Statement**
To ensure full reproducibility of this systematic review and meta-analysis, the complete electronic search strings, Boolean logic, and field restrictions utilized from database inception through February 2026 are detailed below.

**1. Key Search Concepts and Terms**
The search strategy was structurally built around two core concepts combined via the AND operator:

- **Concept 1 (Target Population/Condition):** Terms related to narcolepsy or broad sleep disorders.
- **Concept 2 (Primary Outcome):** Terms related to mortality, death, survival, or fatality.

**2. Database-Specific Search Syntax**

**A. PubMed**

- **Date Filter:** Inception to February 2026
- **Exact Search String:**
  (narcolepsy[MeSH Terms] OR narcolepsy[Title/Abstract] OR narcoleptic[Title/Abstract] OR "sleep disorder"[Title/Abstract]) AND (mortality[MeSH Terms] OR mortality[Title/Abstract] OR death[Title/Abstract] OR survival[Title/Abstract] OR fatal[Title/Abstract] OR "all-cause mortality"[Title/Abstract] OR "cause-specific mortality"[Title/Abstract] OR "survival analysis"[Title/Abstract])

**B. Embase (via Elsevier)**

- **Date Filter:** Inception to February 2026
- **Exact Search String:**
  ('narcolepsy'/exp OR 'narcolepsy':ti,ab OR 'narcoleptic':ti,ab OR 'sleep disorder':ti,ab) AND ('mortality'/exp OR 'mortality':ti,ab OR 'death':ti,ab OR 'survival':ti,ab OR 'fatal':ti,ab OR 'all-cause mortality':ti,ab OR 'cause-specific mortality':ti,ab OR 'survival analysis':ti,ab)

**C. Scopus**

- **Date Filter:** Inception to February 2026
- **Exact Search String:**
  (TITLE-ABS-KEY(narcolepsy) OR TITLE-ABS-KEY(narcoleptic) OR TITLE-ABS-KEY("sleep disorder")) AND (TITLE-ABS-KEY(mortality) OR TITLE-ABS-KEY(death) OR TITLE-ABS-KEY(survival) OR TITLE-ABS-KEY(fatal) OR TITLE-ABS-KEY("all-cause mortality") OR TITLE-ABS-KEY("cause-specific mortality") OR TITLE-ABS-KEY("survival analysis"))

**D. Web of Science (Core Collection)**

- **Date Filter:** Inception to February 2026
- **Exact Search String:**
  TS=(narcolepsy OR narcoleptic OR "sleep disorder") AND TS=(mortality OR death OR survival OR fatal OR "all-cause mortality" OR "cause-specific mortality" OR "survival analysis")

**3. Manual Screening and Grey Literature**
In addition to the electronic database strings above, the reference lists of all successfully retrieved full-text articles, as well as relevant narrative reviews published within the last 5 years, were hand-searched to identify any missing or uncaught cohort studies. No language restrictions or specific demographic filters were applied during the initial execution of these search configurations.
